# Osteopontin: a central hub in the pathogenesis and therapeutic intervention of liver disease

**DOI:** 10.1080/07853890.2025.2596538

**Published:** 2025-12-12

**Authors:** Junran Yang, Yufei Yao, Zhenhua Zhou

**Affiliations:** ^a^ Cellular Immunology Laboratory, Shanghai University of Traditional Chinese Medicine Affiliated Shuguang Hospital, Shanghai, China; ^b^ Department of Hepatology, Shanghai University of Traditional Chinese Medicine Affiliated Shuguang Hospital, Shanghai, China

**Keywords:** Osteopontin, therapeutic target, pathogenesis, macrophage, molecular mechanism

## Abstract

**Background:**

Osteopontin (OPN) is a phosphorylated glycoprotein implicated in inflammation and tissue remodeling. Its expression is significantly elevated in various liver diseases, but its precise pathophysiological roles remain complex and context-dependent.

**Objective:**

This review systematically examines the mechanisms of OPN in multiple liver diseases, including acute liver injury, alcoholic liver disease, viral hepatitis, metabolic-associated fatty liver disease, and hepatocellular carcinoma. It focuses on its cell-type-specific functions and explores its potential as a diagnostic biomarker and therapeutic target.

**Results:**

OPN exhibits a dual role in liver pathophysiology. It promotes disease progression by activating hepatic stellate cells to drive fibrosis, enhancing collagen deposition, and facilitating HCC invasion and metastasis. Conversely, OPN also demonstrates protective functions. Intestinal OPN preserves gut barrier integrity and microbiome homeostasis, ameliorating alcohol-induced liver injury. Notably, a recent identified mechanism reveals that macrophage-derived OPN activates the OSM–STAT3–ARG2 signaling axis in hepatocytes, enhancing fatty acid oxidation and attenuating hepatic steatosis in MAFLD. Furthermore, OPN shows promise as a clinical biomarker for detecting early-stage HCC and assessing liver fibrosis, potentially outperforming alpha-fetoprotein.

**Conclusion:**

Osteopontin serves as a central signaling hub in liver diseases, where it effectively integrates inflammatory, metabolic and fibrogenic networks. Harnessing its therapeutic potential represents the cornerstone for developing future OPN-targeted therapies. This strategic approach will create new avenues for liver disease treatment, enabling precise interventions tailored to specific disease contexts.

Osteopontin (OPN), also known as bone salivary protein 1 (BSP-1), is a highly phosphorylated glycoprotein increasingly recognized for its role in the progression of tissue fibrosis. OPN functions as a secreted cytokine and an extracellular matrix protein, playing pivotal roles in inflammation, immune regulation, and wound healing. As a key immunomodulatory molecule, OPN plays a vital role in numerous physiological and pathological processes critical to tissue remodeling [1]. In recent years, osteopontin (OPN) has been demonstrated to be extensively involved in the progression of various liver diseases, including acute liver failure (ALF) [2,3], metabolism-associated fatty liver disease(MAFLD) [4,5], alcoholic liver disease(ALD) [6] and associated with chronic hepatitis B (CHB) [7] or chronic hepatitis C (CHC) [8] liver fibrosis. Moreover, studies have shown that OPN can serve as a diagnostic marker for hepatocellular carcinoma (HCC), with its sensitivity potentially surpassing that of traditional liver cancer marker alpha-fetoprotein (AFP) [9]. This review systematically examines the mechanisms of osteopontin (OPN) in various liver diseases—including acute liver injury, alcoholic liver disease, viral hepatitis, metabolic-associated fatty liver disease, and hepatocellular carcinoma—with a particular focus on its cell-type-specific functional roles and associated signaling networks. It further explores the translational potential of OPN as a diagnostic biomarker and therapeutic target, aiming to provide a theoretical foundation for precision medicine in hepatology.

## 1. The physiological role of OPN in the body

OPN is a multifunctional phosphorylated glycoprotein that plays a key role in various physiological and pathological processes. In the skeletal system, OPN mediates osteoclast adhesion and regulates bone mineralization through its RGD domain, and its phosphorylation state directly affects the formation of hydroxyapatite crystals [10,11]. In terms of immune regulation, OPN is secreted by T cells, macrophages, and others, activating signaling pathways through integrins and CD44 receptors, regulating Th1/Th17 cell differentiation, macrophage function, and the release of pro-inflammatory factors (such as IL-12, TNF-α), thereby influencing inflammation and autoimmune responses [12–14]. In intestinal health, OPN enhances the expression of tight junction proteins (such as ZO-1, Claudin-4), maintains the integrity of the intestinal barrier, and optimizes the gut microbiota by regulating short-chain fatty acids (SCFA) and bile acid metabolism [6,15]. In addition, OPN is involved in neurodevelopment and cognitive function, promoting the growth of brain regions, neuron survival, and synaptic plasticity, and plays a neuroprotective role after brain injury [16–18]. Its wide-ranging functions rely on post-translational modifications such as phosphorylation and glycosylation, which play important roles in tissue repair, cancer progression, and metabolic regulation [19,20].

## 2. The role of OPN in liver diseases

### 2.1. The role of OPN in the onset of ALF

In the pathogenesis of acute liver failure (ALF), OPN plays a complex dual role: on one hand, OPN levels are related to the degree of liver necrosis in acute liver injury [21], It significantly exacerbates the local inflammatory response by attracting neutrophils, lymphocytes, and macrophages to infiltrate the damaged area of the liver [22]; On the other hand, studies have also shown that OPN can upregulate the acetylation and translocation of high mobility group box 1 (HMGB1), thereby promoting the production of collagen by hepatic stellate cells and driving the progression of liver fibrosis [23]. Clinical observations have found that in patients with acute liver failure (ALF) caused by drug-induced liver injury (DILI), the downregulation of plasma OPN levels is closely related to poor prognosis, including death or the need for liver transplantation [3]. This indicates that the dynamic changes of OPN can serve as an important inflammatory biomarker for assessing the severity and prognosis of ALF.

### 2.2. The role of OPN in the onset of ALD

In alcohol-associated liver disease (ALD), OPN expression is markedly increased, and its plasma levels correlate positively with disease severity [24,25]. Mechanistically, OPN drives hepatic stellate cell (HSC) activation—thereby accelerating liver fibrosis—by stimulating the hedgehog pathway [26] and enhancing phosphorylation of Akt and Erk [25]. Furthermore, OPN upregulates type I collagen synthesis by suppressing microRNA miR-129-5p, a negative regulator of collagen expression [27]. These findings collectively indicate that OPN acts as a key pro-inflammatory and pro-fibrotic mediator in ALD.

Conversely, intestine-derived OPN exerts protective effects against alcohol-induced liver injury by preserving gut microbial homeostasis and barrier integrity [28]. Specifically, overexpression of OPN in intestinal epithelial cells (IECs) or exogenous administration of milk-derived OPN induces antimicrobial peptides that help maintain microbial community structure. OPN also enhances tryptophan metabolite and short-chain fatty acid production, strengthening gut barrier function *via* the aryl hydrocarbon receptor (Ahr) pathway and ameliorating ALD. However, some studies report that OPN deficiency does not uniformly confer protection. In a model of alcoholic neutrophilic hepatitis, OPN knockout promoted hematopoietic stem cell mobilization to the liver and increased iron accumulation, exacerbating liver injury [29]. Other investigations have demonstrated protective roles of OPN in mouse models of ALD [30], highlighting context-dependent functions.

### 2.3. The role of OPN in the onset of viral hepatitis

OPN has been identified as a critical mediator in the progression of hepatitis B virus (HBV) infection, with its expression being regulated by increased matrix stiffness during liver fibrosis. Under *in vitro* culture conditions mimicking fibrotic stiffness (24 kPa), OPN expression was significantly upregulated in HBV-transfected HepG2.2.15 cells. This upregulation was accompanied by suppression of interferon-stimulated genes (ISGs), including OAS1, ISG15, and APOBEC3G, as well as reduced phosphorylation of STAT1, ultimately leading to increased levels of HBV RNA, DNA, and HBsAg [31]. These findings align with clinical observations showing elevated serum OPN levels in patients with CHB and HCC [32]. OPN has also been recognized as a biomarker for liver fibrosis [33]. Further mechanistic studies revealed that OPN upregulates the protein expression of USP18, a negative regulator of interferon signaling, thereby suppressing the type I interferon pathway, impairing innate immune responses, and promoting HBV replication [34]. In a CCl_4_-induced liver fibrosis model using HBV transgenic mice, increased liver stiffness correlated with higher OPN levels, downregulation of ISGs, and elevated HBV markers, supporting the role of OPN in promoting HBV infection within a fibrotic microenvironment *via* suppression of antiviral immunity [31].

In the context of CHC, studies suggest that serum OPN levels may serve as a non-invasive marker for assessing liver fibrosis, effectively predicting the severity of fibrotic progression in CHC patients [8].

### 2.4. The role of OPN in the pathogenesis of MAFLD

Based on previous assessments of OPN’s translational potential as a biomarker and therapeutic target in liver disease, it is now recognized that unhealthy dietary patterns and alcohol consumption act synergistically to drive the onset and progression of MAFLD by inducing hepatic lipid metabolic dysregulation, oxidative stress, and inflammation [35,36]. Against this backdrop, elucidating the central role of OPN in MAFLD has become increasingly critical. OPN contributes to the progression of MAFLD by suppressing autophagy and promoting hepatocyte senescence [37]. In the liver, autophagy plays vital roles in lipid metabolism, cellular repair, and anti-inflammatory responses. As an essential degradative pathway, autophagy removes damaged organelles, protein aggregates, and excess lipid droplets, thereby maintaining cellular homeostasis. Impairment of autophagic flux prevents the effective clearance of accumulated lipids and harmful substrates, leading to heightened oxidative stress. Concurrently, the accumulation of senescent hepatocytes exacerbates local inflammation, which further inhibits autophagy recovery. This vicious cycle promotes persistent hepatic inflammation and fibrosis, potentially progressing to cirrhosis and hepatocellular carcinoma. Extracellular OPN promotes obesity and modulates lipid synthesis [38]. In diet-induced obesity models [39], OPN knockout or neutralization reduces adipose tissue inflammation and insulin resistance, indicating that extracellular OPN contributes to metabolic syndrome and steatosis. Administration of recombinant OPN in wild-type mice increases hepatic cholesterol content, suggesting a role for OPN in regulating bile acid metabolism and de novo lipogenesis. While OPN knockout mice fed a high-fat diet (HFD) develop less steatosis, streptozotocin-induced diabetic OPN knockout models exhibit increased hepatic triglycerides under HFD feeding [37]. Clinically, serum OPN correlates positively with hepatic triglycerides and cholesterol in non-obese MAFLD patients, but negatively in obese patients [40], underscoring the context-dependent mechanisms of OPN in liver and adipose tissue.

These findings imply that increased OPN expression in macrophages infiltrating the liver may be linked to enhanced macrophage activity, offering new insight into MAFLD pathogenesis. Interestingly, recent evidence suggests macrophage-derived OPN may attenuate MAFLD progression, indicating a potential therapeutic target worthy of further investigation.

Oncostatin M (OSM), a pleiotropic cytokine of the IL-6 family, is produced by activated monocytes/macrophages, T cells, dendritic cells, and neutrophils [41]. Beyond its roles in inhibiting tumor growth and promoting differentiation, OSM is involved in inflammation, hematopoiesis, and tissue repair. Arginase 2 (ARG2), the final enzyme in the urea cycle, catalyzes the conversion of L-arginine to L-ornithine and urea. Dysregulation of the urea cycle has been associated with MAFLD progression [42]. In mouse models, ARG2 upregulation correlates with reduced blood triglycerides, and ARG2 overexpression lowers hepatic TG in HFD-fed mice [43].

Notably, OPN does not directly affect ARG2 expression but induces OSM generation through transactivation of αvβ3 integrin and PDGFR in primary osteoblasts [44]. OPN recruits macrophages and neutrophils to the liver, induces IL-6 production, and promotes STAT3 activation in hepatocytes [45]. OSM activates intracellular signaling pathways that upregulate IL-6 transcription, amplifying inflammatory responses such as acute-phase protein production. Through STAT3 signaling in hepatocytes, macrophages enhance ARG2 expression. Increased ARG2-mediated fatty acid oxidation (FAO) alleviates steatosis. Therefore, enhancing the OPN–OSM–STAT3–ARG2 axis between macrophages and hepatocytes may confer benefits in MAFLD [46], identifying this pathway as crucial for limiting lipid accumulation in hepatocytes during disease progression.

### 2.5. The application of OPN in HCC

OPN contributes to the pathogenesis of HCC through multiple mechanisms. By engaging with integrins and CD44 receptors, OPN enhances hepatocyte growth factor (HGF)-induced cellular scattering and invasion, and activates the c-Met signaling pathway, thereby facilitating HCC progression. Additionally, OPN binds to vimentin and stabilizes it by inhibiting its degradation, which promotes epithelial–mesenchymal transition (EMT) and increases tumor cell migration and invasiveness  [47–49]. In inflammatory liver diseases, OPN acts as a chemotactic factor for macrophages and neutrophils, further shaping the tumor microenvironment [49]. OPN is an integrin-binding glycoprotein widely expressed in various tissues and is highly overexpressed in HBV-associated HCC. It drives cancer progression by promoting metastasis, modulating cellular metabolism, and participating in both tissue repair and carcinogenic processes [50]. Although OPN serves as a marker for biliary epithelial cells (BECs) and hepatic progenitor cells (HPCs), and a small number of OPN-positive cells contribute to liver regeneration after certain chronic injuries [51,52], lineage-tracing studies in multiple toxin-induced HCC models indicate that OPN-labeled BECs/HPCs do not directly give rise to tumors, suggesting that hepatocytes are the primary cell origin of HCC under these conditions  [53,54].

Notably, in HBV-related HCC, elevated circulating OPN levels are closely associated with intrahepatic metastasis and early tumor development [55]. OPN expression is significantly higher in HBsAg-positive individuals and demonstrates high sensitivity (97%), specificity (70%), and overall accuracy (84%) as a biomarker. Serum OPN also shows potential in predicting responses to anti-PD-L1 immunotherapy [56], underscoring its value as a prognostic indicator for HCC progression and patient outcomes [32,50, 57,58]. Moreover, OPN outperforms alpha-fetoprotein in the detection of early-stage HCC  [59].

### 2.6. Role of OPN in liver fibrosis

OPN plays a central role in the development of liver fibrosis [60] through multiple mechanisms that activate hepatic stellate cells (HSCs) and promote collagen deposition. Following liver injury, OPN is secreted by hepatocytes and inflammatory cells, and activates the PI3K/pAkt/NFκB pathway—leading to upregulated expression of type I collagen and accelerating fibrogenesis [23,61].

Growing experimental evidence indicates a strong association between OPN and fibrosis in MAFLD [62,63]. Studies suggest that OPN facilitates intercellular communication between hepatocytes expressing E4BP4 and HSCs, promoting fibrogenesis in MAFLD  [64]. Furthermore, OPN stimulates cholangiocytes to secrete chemokines and recruits pro-inflammatory monocytes, enhancing macrophage accumulation and accelerating fibrosis in MAFLD [65]. These findings highlight OPN as a key molecular driver of liver fibrosis and a potential therapeutic target. The key mechanisms of OPN in the liver diseases discussed above are summarized (Table 1).

**Table 1.** Summary of the mechanisms of OPN in liver diseases.

Liver disease	Model	Effect	Action target	Molecular mechanism	References
ALD	LX2 cell line	Deleterious	Akt- signal pathway, Erk-signal pathway, Hedgehog-signal pathway	OPN↑→CD44v6/αvβ3 Integrin, P-Akt, Hepatic Stellate Cell Activation↑	[25, 26]
	LX2 cell line	Deleterious	miR-129-5p signal pathway	OPN↑→miR-129-5p level↓→ Collagen↑	[27]
	OPN knockout C57BL/6J mice	Positive	Ahr	OPN↑→IECs tryptophan metabolites ↑→ AHR-short-chain fatty acid↑	[28]
HBV	HBV-transfected HepG2.2.15 cells	Deleterious	OAS1, ISG15, APOBEC3G	OPN↑→ISGs (OAS1, ISG15, APOBEC3G) ↓/STAT1 →HBV RNA, DNA↑	[31]
	HBV transgenic mice	Deleterious	STAT1, ISGs	OPN↑→STAT1/ISG↓→HBV replication↑	[31]
MAFLD	OPN knockout C57BL/6J mice + HFFC Diet/Mouse Macrophage	Positive	αvβ3 integrin, PDGF, OSM, IL-6, STAT3, ARG2	OPN↑→αvβ3 integrin/PDGFR →OSM →IL-6↑→STAT3 →ARG2↑→FAO ↓	[46]
HCC	Mouse hepatocytes	Deleterious	Integrin, CD44, HGF, c-Met	OPN↑→integrins/CD44 → c-Met; OPN↑→ EMT↑	[58]
Liver fibrosis	Mouse hepatic stellate cells	Deleterious	Integrin, αvβ3, PI3K, AKT, NFκB	OPN↑→ integrin, αvβ3/PI3K/AKT/NFκB↑ → Collagen-I↑	[23]

## 3. Discussion

**Figure 1.**
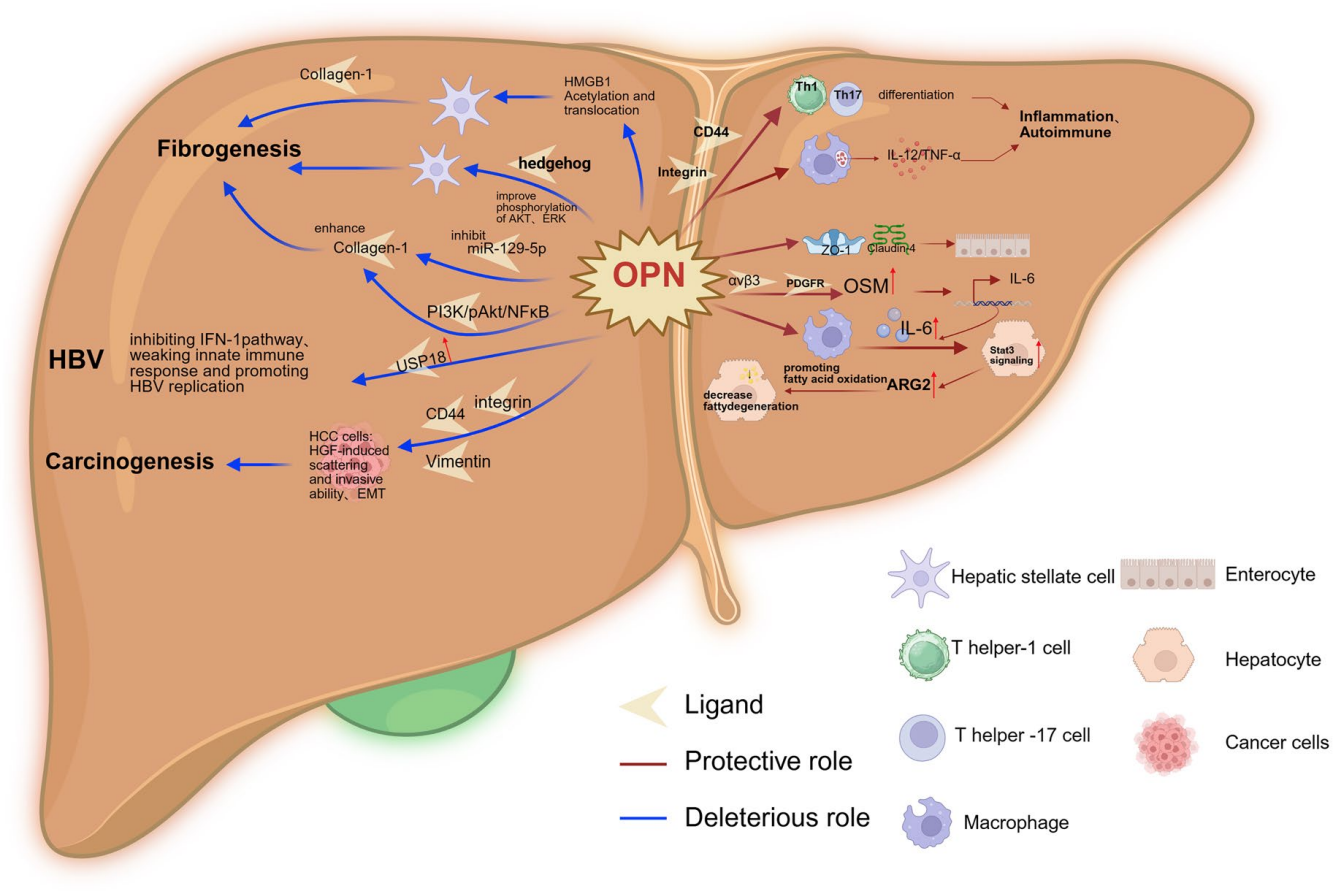
The dual roles and mechanisms of OPN in liver pathophysiology.

This article systematically elaborates the central role of OPN in various liver diseases, highlighting its functional complexity and context-dependent nature (Figure 1). The multifaceted role of osteopontin (OPN) in liver pathophysiology is shaped by its cellular origin, spatiotemporal expression, and disease-specific microenvironment. While this review outlines the well-established roles of OPN in promoting fibrosis and tumorigenesis, it also highlights its emerging protective functions—particularly in preserving gut–liver axis integrity and modulating metabolic pathways—thereby broadening our understanding of this multifunctional molecule. A central unanswered question remains the molecular mechanisms underpinning OPN’s functional duality, which represents a crucial step toward harnessing its therapeutic potential. The recently identified OPN–OSM–STAT3–ARG2 axis exemplifies this complexity. Macrophage-derived OPN can enhance fatty acid oxidation in hepatocytes *via* this signalling cascade, paradoxically attenuating steatosis. This finding challenges the conventional view of OPN solely as a pathological mediator and emphasizes the importance of cellular context. OPN’s functional effects are closely tied to its cellular source (e.g. hepatocytes, macrophages, cholangiocytes, or intestinal epithelial cells), receptor specificity (such as αvβ3 integrin vs. CD44 variants), and post-translational modifications. Commonly used global OPN-knockout models fail to capture this cell-specific functional diversity, potentially exaggerating detrimental roles while overlooking protective functions.

Notably, oxidative stress plays a critical role in liver pathophysiology: moderate oxidative eustress can activate endogenous protective mechanisms and enhance hepatic tolerance to ischemia-reperfusion injury, whereas excessive oxidative distress serves as a key pathogenic basis for various liver diseases [66]. Furthermore, osteopontin (OPN) and oxidative stress engage in a self-amplifying vicious cycle. As highlighted in this review, OPN functions as an oxidative stress-sensitive cytokine whose expression is upregulated by oxidative stress. The elevated OPN, in turn, exacerbates oxidative stress by suppressing protective autophagy [37]. This positive feedback loop is likely to play a central driving role in the development and progression of persistent inflammation and liver fibrosis. This contextual dependency helps reconcile contradictory observations in the literature. For instance, in alcoholic liver disease (ALD), intestinal OPN exerts systemic protection by maintaining microbial homeostasis and barrier function, whereas hepatic OPN directly activates stellate cells and propagates inflammation. Thus, the efficacy of OPN-targeted therapies will depend on precise cellular or molecular targeting. A major current limitation is the lack of tools capable of selective inhibition of OPN from specific cellular sources or isoforms at particular disease stages.

OPN demonstrates multifaceted clinical value in hepatic diseases. It serves not only as a significant biomarker for early hepatocellular carcinoma screening, liver fibrosis assessment, and immunotherapy response prediction, but also reveals novel therapeutic dimensions through recently identified mechanisms—specifically, macrophage-derived OPN alleviates hepatic steatosis *via* activation of the OSM–STAT3–ARG2 signaling axis. Furthermore, the functional dichotomy of OPN derived from different cellular sources, such as its protective role in the gut versus its injurious effects in the liver, offers promising avenues for developing cell-specific targeted therapies and personalized monitoring strategies.

Given this broad spectrum of potential applications, translating OPN into clinical practice necessitates refined and targeted approaches. Broad OPN inhibition risks disrupting its homeostatic roles in tissues such as the gut and bone. Future efforts should focus on: (1) developing antagonists that selectively block OPN binding to pro-fibrotic receptors (e.g. αvβ3); (2) employing nanocarriers to deliver OPN-silencing agents specifically to stellate cells or tumour-associated macrophages; and (3) leveraging OPN’s value as a biomarker—its elevated expression in HBV-related hepatocellular carcinoma correlates with metastasis and response to immunotherapy, supporting its use in patient stratification, treatment monitoring, and early diagnosis.

## 4. Conclusion

OPN is neither a simple villain nor a hero in liver disease, but a sophisticated central hub that integrates inflammatory, metabolic, and fibrogenic signals. Its dual role is a feature, not a bug, reflecting its evolutionary role in coordinating wound healing and tissue remodeling. The next frontier of OPN research requires moving from association to causation, utilizing sophisticated tools such as cell-specific knockout models, single-cell omics, and spatial transcriptomics to map its spatiotemporal functions with high precision. By embracing this complexity, we can shift the goal from blanket inhibition to context-aware modulation, ultimately paving the way for OPN-based precision medicine in hepatology.

## Data Availability

There is no data associated with this research.
